# Sanhuang Decoction Controls Tumor Microenvironment by Ameliorating Chronic Stress in Breast Cancer: A Report of Ninety Cases

**DOI:** 10.3389/fonc.2021.677939

**Published:** 2021-08-17

**Authors:** Ming Feng, Huanhuan Wang, Zhiyuan Zhu, Bowen Yao, Yongfei Li, Jingxian Xue, Sihan Cao, Xinyi Shao, Yanlei Xu, Ki Cheul Sohn, Im Hee Shin, Chang Yao

**Affiliations:** ^1^The First Clinical college, Nanjing University of Chinese Medicine, Nanjing, China; ^2^Department of Breast Disease, Affiliated Hospital of Nanjing University of Chinese Medicine, Nanjing, China; ^3^Sidney Kimmel Medical College, Thomas Jefferson University, Philadelphia, PA, United States; ^4^School of Medicine, Catholic University of Daegu, Gyeongsan, South Korea

**Keywords:** breast cancer, endocrine treatment, chronic stress, Sanhuang decoction, SHD, body microenvironment

## Abstract

Long-term endocrine treatment which results in estrogen deprivation causes chronic stress associated with a series of uncomfortable symptoms leading not only to a decrease in quality of life but also to cancer recurrence, which may be mediated primarily through the enhanced expression of angiogenic factors, as well as a series of inflammatory microenvironmental changes that favor tumor progression. In this study, we designed a clinical trial and aimed to explore the effects of Sanhuang Decoction (SHD) treatment on chronic stress, inflammatory factors, and breast cancer recovery. A total of 90 patients with breast cancer who met the inclusion/exclusion criteria were randomly allocated to a treatment or control group. The treatment group received the standard endocrine treatment and the traditional Chinese medicine decoction known as SHD. The control group received the standard endocrine treatment only. The treatment period was 6 months. The modified Kupperman Menopausal Index, the self-rating anxiety scale, and the self-rating depression scale were evaluated once per month. The body microenvironment plasma indices related to chronic stress, such as oxidative and antioxidative stress markers, inflammatory factors, hemorheology, coagulation, lipid and D-dimer, immunologic functions, tumor biomarkers, and angiogenic factors of the vascular endothelial growth factor (VEGF) were measured before and after 6 months of treatment. After treatment for 5 months, the scores in the treatment group decreased to nearly normal levels and the control group showed no significant improvement. After treatment for 6 months, all indices related to the body microenvironment, as well as the tumor biomarkers and carcinoembryonic antigen, carbohydrate antigen 153, and angiogenic factor VEGF levels improved significantly to normal levels in the treatment group. Our primary research showed that treatment with SHD effectively improved the quality of life of breast cancer patients by facilitating a change in the body microenvironment that controlled tumor growth and prevented drug resistance.

**Clinical Trial Registration:**

Chinese Clinical Trial Registry, identifier ChiCTR-IIR-2000041413. Date of registration: 2017-06-07 (retrospective registration).

## Introduction

Breast cancer is the most common cancer among Chinese women (1). Breast cancer treatments have evolved over the last 40 years from profound and aggressive to conservative procedures that minimize tissue trauma and physical deformity. This is because the overall survival has improved with developments in surgery, chemotherapy, radiotherapy, target therapy, endocrine therapy, and so on. Among these, the endocrine treatment lasting 5–10 years is usually considered the standard choice for patients with estrogen receptor-positive cancers for 5 or 10 years (2, 3). However, such a long period of estrogen deprivation caused by the administration of selective estrogen receptor modulators or aromatase inhibitors results in chronic stress with symptoms of anxiety, poor appetite, difficulty falling asleep, and arthralgia syndrome; all of which not only lead to a decrease in the quality of life but also induce the recurrence of cancer (4). Studies have reported that chronic stress can alter immunological, neurochemical, and endocrine functions leading to cancer progression, which may be mediated primarily through the activation of the tumor cell phosphoinositide 3‐kinase (PI3K) signaling pathway. This results in a markedly increased vascularization and an enhanced expression of the vascular endothelial growth factor (VEGF), matrix metalloproteinase (MMP)-2, and MMP-9 (5, 6).

When exposed to stress over a long period of time, people produce a series of inflammatory microenvironmental changes that favor tumor progression (7). Studies have demonstrated that oxidative stress increases the levels of proinflammatory cytokines, such as tumor necrosis factor (TNF)-α and interleukin-6 (IL-6), and upregulates inflammatory molecules, such as vascular cell adhesion molecule-1, intercellular adhesion molecule-1, and nuclear factor-kappa B (NF-кB); all of which contribute to tumor growth, migration, and metastasis (8).

Researchers have reported many different effects of acute and chronic oxidative stress on tumor growth. One study showed that the growth of estrogen receptor-positive breast cancer cells (MCF-7) was inhibited during acute exposure to an oxidative stress environment, whereas exposure to such a sustained chronic environment over a period of 3 months promoted a significant growth. The researchers further analyzed the related gene expression and found an upregulation of the pro-metastatic genes, VEGF, WNT1, and cluster of differentiation 44 (CD44), and a downregulation of the anti-metastatic gene E-cadherin was observed in cells under persistent exposure to oxidative stress for 3 months (9, 10). Studies have also suggested that while tumor cells adapted to the long-term reactive oxygen species (ROS)-induced toxicity, this promoted acquired multidrug resistance in breast cancer cells through the PI3K/protein kinase B (PI3K/Akt) and NF-ĸB pathways regulated by the stress-related factors, NF-E2-related factor 2 (Nrf2), hypoxia-inducible factor 1, and protein kinase C (11). However, these results are derived from experimental research and have not yet been verified by clinical trials.

Traditional Chinese medicine (TCM) is primarily used as a complementary alternative medicine. TCM has a history of more than 2,500 years in China and is built on the view that the body is a series of functional entities, rather than looking at the fixed somatic structures that perform the activities. It focuses on harmonizing the inner environment of the body with its natural surroundings using tools including herbal medicine, acupuncture, massage, qigong, and dietary modifications, among others, and herbal medicines remain the principal tool in China (12).

Most breast cancer patients experience symptoms of fatigue, anxiety, fixed incision pain, loss of appetite, lusterless complexion, pale tongue, and weak pulse, and they are treated with long-term endocrine therapy. All of the abovementioned symptoms are manifestations of stress (13, 14). The TCM decoction known as Sanhuang Decoction (SHD) is a type of herbal medicine composed of astragalus, turmeric, and rhubarb, which are tonic substances that improve the immune system to relieve fatigue and promote blood circulation (15). SHD is composed of Dahuang, Huangqi, and Jianghuang. *Rhei Radix et Rhizoma* (rhubarb, known as Dahuang in Chinese), belongs to the *genus Rheum L*. in the *Polygonaceae* family, and it is composed of dried roots and rhizomes, including *Rheum palmatum* L., *Rheum tanguticum* (Maxim. *ex Balf*.), and *Rheum officinale Baill*. Rhubarb plays a role in many pharmacological activities, such as purgation, anti-inflammation, anti-cancer, and hepatoprotection, and has positive benefits on the gallbladder. *Astragali Radix* (AR) (known as Huangqi in Chinese) is one of the most popular herbal medicines used worldwide. It is the dried root of *Astragalus membranaceus (Fisch.) Bg*, or *Astragalus membranaceus (Fisch.) Bge.* var.*, mongholicus (Bge.) Hsiao*. This herb possesses tonic, hepatoprotective, diuretic, and expectorant properties (Chinese Pharmacopoeia Commission, 2010) (16) and has been shown to exhibit immunomodulatory, antihyperglycemic, anti-inflammatory, antioxidant, and antiviral activities, among others. Traditionally, it has been used to treat weakness, wounds, anemia, fever, multiple allergies, chronic fatigue, loss of appetite, uterine bleeding, and uterine prolapse. The rhizome of *Curcuma Longa*, turmeric (Jianghuang in Chinese), is used as a Chinese medicine which can expel gas line and pass through the pain, and the selected prescription takes the power of fried astragalus to invigorate qi, which helps rhubarb to activate blood circulation and turmeric to push qi. When qi is sufficient, it increases movement and removes dampness and stasis. It can relieve dyspnea, hypodynamia, and spontaneous perspiration, as well as alleviating symptoms, such as cumbrous, anesthesia caused by phlegm, dampness, and blood stasis. In China, SHD has been applied in clinical trials to treat breast cancer in combination with chemotherapeutic drugs (17–22). Our previous clinical research results showed that SHD could not only ameliorate the symptoms but also the serological microenvironments of stress during the perioperative period. In our previous studies, we found that SHD reduces oxidative stress and suppresses tumor angiogenesis by inhibiting the PI3K and aurora kinase signaling pathways. We also demonstrated the synergetic and anti-resistant effects of SHD with endocrine therapy on breast cancer (23). Therefore, it is reasonable to further explore the potential effects of SHD on chronic stress with long-term endocrine therapy.

Numerous clinical trials have focused on controlling chronic stress in breast cancer patients with a series of psychological interventions and comprehensive lifestyle changes, such as prescribed exercise programs, mindfulness-based stress reduction, and bioactive natural dietary supplements. Primary conclusions have revealed that patients benefit from a positive attitude toward chronic stress, leading to relief of insomnia, anxiety, and fatigue with an enhanced quality of life (24). It was previously reported that psychological interventions could significantly reduce the risk of breast cancer recurrence and death from breast cancer (P = 0.034 and P = 0.016, respectively) (25). However, such trials focused on the improvement of the subjective feelings of the patient and did not report on any objective evaluations related to tumor changes.

Several studies have suggested that oxidative stress, chronic inflammation, and cancer are closely linked. Studies have found that long-term stress generated ROS that recruit inflammatory cells and stimulate tumor progression and recurrence (26). Therefore, in this study, we designed a clinical trial and aimed to explore the effects of SHD treatment on chronic stress, inflammatory factors, and breast cancer recovery.

## Methods

### Methods and Study Protocol

The research protocol was approved by the Institutional Review Board of Human Research at the Affiliated Hospital of Nanjing University of Traditional Chinese Medicine. Ninety patients from the Department of Breast Diseases, Jiangsu Provincial Hospital of TCM, were identified, screened, and enrolled in the study between June 2017 and December 2018, and all patients provided an informed consent.

### Inclusion/Exclusion Criteria

Patients in this study were recruited from a population of patients undergoing endocrine treatment and were invited to participate in the study if they fulfilled all of the following criteria: 1) a diagnosis of breast cancer with planned modified radical surgery and 2) between the ages of 30 and 70 years. Patients were included in the study if they: 1) provided informed consent; 2) showed estrogen receptor and/or progesterone receptor positivity (by pathological immunohistochemical detection); and 3) underwent more than 6 months of endocrine treatment. Patients were excluded if they had: 1) inflammatory breast cancer; 2) central nervous system metastases; 3) symptomatic visceral disease; 4) clinically significant, uncontrolled heart disease; or 5) a cardiac repolarization abnormality, including a QT interval corrected for heart rate according to Frederica’s formula greater than 450 ms; 6) recurrence or metastasis diagnosed by imaging or histology;7) participating in other clinical trials; and 8) in pregnancy or lactation or accompanied by severe diabetes.

If a patient withdrew consent, failed to adhere to the research protocol, or experienced a serious adverse event, they were recorded as withdrawn and not included in the analysis.

Written informed consent was obtained from all the enrolled patients. The trial was performed in accordance with the Good Clinical Practice guidelines and the Declaration of Helsinki. The study protocol and any amendments were approved by an independent ethics committee or institutional review board at each site (IRB application receipt 2017NL-007-03). A study steering committee comprising participating international investigators and Novartis representatives oversaw the execution of the trial. An independent data monitoring committee assessed the safety data.

### Study Design

Ninety female patients were randomly and prospectively assigned (1:1) to the treatment group or control group by permuted block randomization through interactive response technology. At enrolment, investigators registered patients in the interactive response technology system with their identifying information, and then, the patients were assigned a seven-digit number that was retained throughout their participation in the study to facilitate anonymity. All patients, and investigators administering treatment, assessing outcomes, and analyzing data were masked to the treatment group assignment. Masking to group assignment was ensured with the use of matching placebos with identical packaging, labeling, schedule of administration, and appearance. The sponsor was masked to the randomized treatment group allocation.

### Intervention

All patients were treated with the standard endocrine treatment with or without ovarian function suppression according to the breast cancer guidelines of the Chinese Society of Clinical Oncology. In the treatment group, the patients received 100 mL of SHD solution twice daily for six consecutive months.

SHD containing 30 g of *Astragalus membranaceus* (TCM name: Huang qi), 10 g of *Rheum officinale* (TCM name: Dahuang), and 10 g of *Curcuma Longa* (TCM name: Jianghuang) were acquired from Jiangsu Province Hospital of TCM (Nanjing, China). The total weight of the crude herb was 50 g. The herbs were blended in 400 mL of double-distilled water (1:8, w/v) for 1 h and heated to 100°C for 2 h. After continuous boiling for 2 h, the remainder of the sample was condensed to 200 mL. The dose was equated to 200 mL SHD daily for an average adult with a body weight of 60 kg. The preparatory steps were completed using a Tisanes device at Jiangsu Province Hospital of TCM. The final 200 mL decoction was administered orally as a split dose twice daily during the entire clinical research period.

### Outcome Measures

#### Measurement Scales

Clinical symptoms focused on chronic stress were measured and recorded once a month for 6 months.

A modified Kupperman Menopausal Index (KMI) was used to measure the quality of life induced by the endocrine treatments. The modified KMI assesses the degree of symptoms, such as hot flushes, sweats, abnormal tactile sensations, insomnia, impatience and ease of irritability, emotional depression, dizziness, fatigue, and aching extremities.

The self-rating anxiety scale (SAS) and self-rating depression scale (SDS) were administered during the endocrine treatment. The SAS and SDS were applied to evaluate the effects of chronic clinical stress in terms of symptoms related to anxiety and depression, respectively.

#### Laboratory Data

Laboratory data were collected for the evaluation of the body microenvironment under chronic stress during the endocrine treatment.

Oxidative and antioxidative stress markers in plasma were measured. Blood samples (5 mL of blood was collected, and blood extracts and sera were prepared for analysis). Serum nitric oxide (NO) (No. A012-1-2; Nanjing Jiancheng Company, Nanjing, China), superoxide dismutase (SOD) (No. A001-3-2; Nanjing Jiancheng Company), malondialdehyde (MDA) (No. A003-1-2; Nanjing Jiancheng Company), glutathione peroxidase (GSH-px) (No. A005-1-2; Nanjing Jiancheng Company), and total antioxidant capacity (TAOC) (No. A015-1-2; Nanjing Jiancheng Company) were measured using a Shimadzu spectrophotometer (Shimadzu Company, Kyoto, Japan). According to the operational instructions, the absorbance value was measured at 550 nm based on a comparison with the standard curve, and the NO and SOD contents were calculated.

Serum levels of TNF-α, IL-6, and IL-8 were evaluated using enzyme-linked immunosorbent assay (ELISA) kits purchased from Beijing 4A Biotech Co., Ltd. (Beijing, China) (Nos. 20150109 and 20150113).

Hemorheology testing, which included whole blood viscosity, plasma viscosity, hematocrit, the erythrocyte aggregation index, and the erythrocyte rigidity index, was undertaken using a blood viscometer (LBY-N6; Beijing Prisheng Instrument Co., Ltd., Beijing, China) with a fasting blood sample. Blood coagulation measures, including prothrombin time, activated partial thrombin time, thrombin time, plasma fibrinogen levels, and D-dimer levels, were measured using an automatic coagulation analyzer (CA-1500; SYSMEX CORPORATION, Kobe, Japan). Lipid indices, including triglyceride (TG), total cholesterol (TC), low-density lipoprotein cholesterol (LDL-C), high-density lipoprotein cholesterol (HDL-C), apolipoprotein-A (apo-A), and apolipoprotein-B (apo-B) levels, were assessed using an automatic biochemical immunity analyzer (Cobas 8000 [Roche Diagnostics GmbH, Mannheim, Germany]; AU5800 [Beckman Coulter, Fullerton, CA, USA]; ZL9600C [Beijing Zhongchi Weiye Technology Development Co., Ltd., Beijing, China]).

Serum levels of CD3, CD4, CD8, CD4/CD8, and immunoglobulin (Ig) G, IgA, IgM, complement 3 (C3), and complement 4 (C4) were detected using a flow cytometry analyzer (FACS Canto II [BD Biosciences, San Jose, CA, USA]; Immage 800 [Beckman Coulter]).

#### Biomarkers

Tumor biomarkers and angiogenic factors of VEGF were evaluated in relation to tumor growth using an ELISA (EK183-96; Lianke Biological Company, Hangzhou, China).

Serum levels of carbohydrate antigen 153 (CA153), CA125, and carcinoembryonic antigen (CEA) were measured using an access immunoassay analyzer (Unicel Dxi800 [Beckman Coulter]; ARCHITECT i2000 SR [Abbott, Abbott Park, IL, USA]).

### Statistical Analysis

The results were analyzed using the SPSS 18.0 (IBM, Armonk, NY, USA). Continuous data were evaluated with a t-test, graded data with a Ridit analysis, and categorical data with an *X^2^* test. Statistical significance was set at P < 0.05.

## Results

### Patient Recruitment and Characteristics

**Figure 1:**
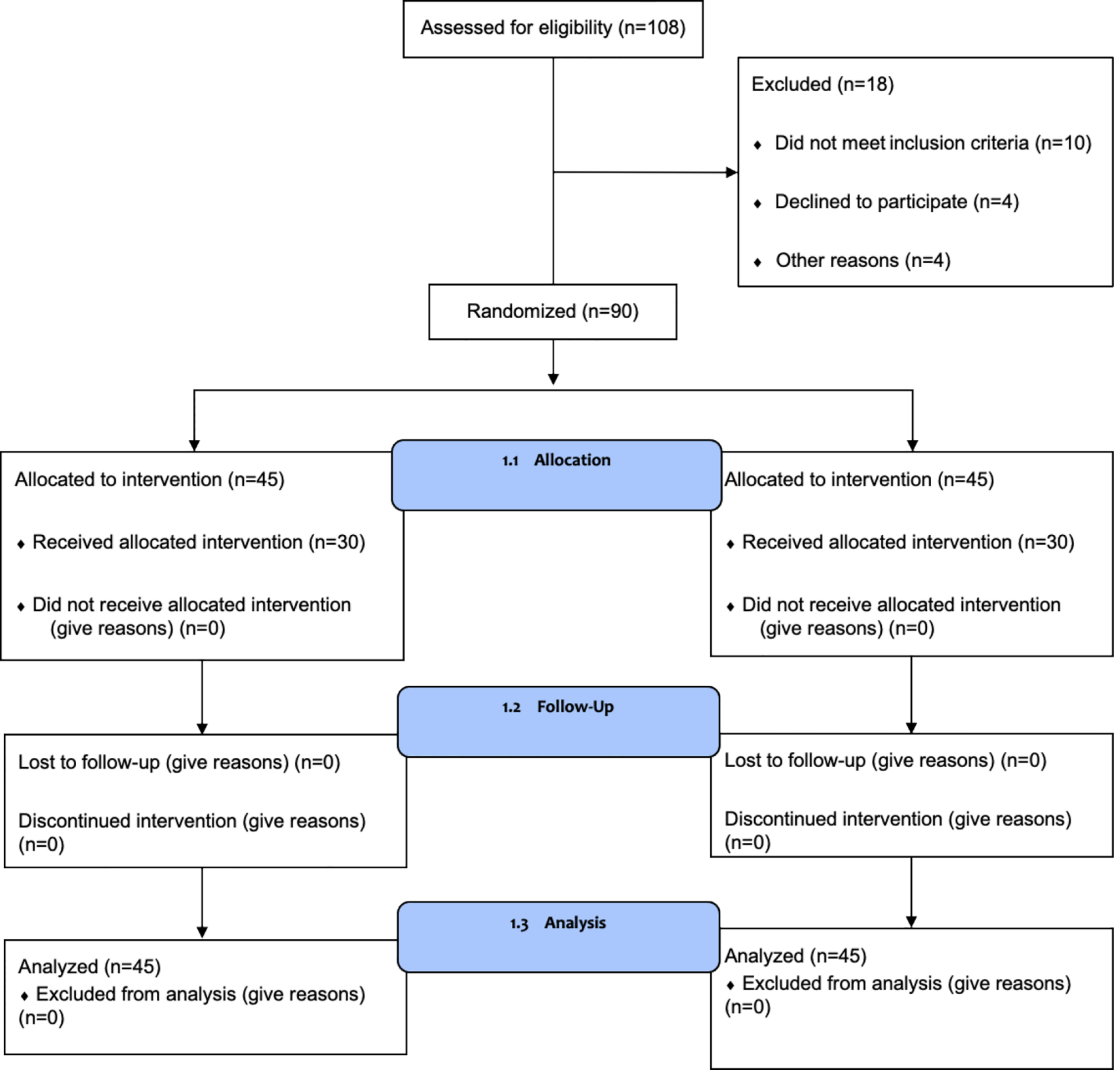
Participant flow chart.

A total of 108 patients were assessed for eligibility, and 90 were enrolled in the study between June 2017 and December 2018. A study flow chart is shown in **Figure 1**. Patients were randomly divided into two groups. There were no statistically significant differences between the two groups in terms of age, clinical stage, modus operandi, types of endocrine treatment, the duration of treatment, and the drugs used for endocrine treatment (**Table 1**).

**Table 1:** Baseline of the subjects.

Index	Experiment group	Control group	P-value
Age (year)	53.8 ± 7.04	51.85 ± 7.84	0.548
Sex	Female (45)	Female (45)	–
TNM staging (n)			0.963
T1N0M0	15	18	
T2N0M0	18	18	
T1N1M0	6	6	
T2N1M0	6	3	
modus operandi			0.755
Simple mastectomy with sentinel lymph node biopsy	30	35	
Radical modified mastectomy	15	10	
Kinds of endocrine treatment			0.855
Tamoxifen	15	18	
Letrozole	12	9	
Anastrozole	12	12	
Exemestane	6	6	
Duration of endocrine treatment			0.724
Half to one year	18	21	
One to one half year	21	15	
One half to two years	6	9	

### Clinical Symptoms Measured by the Modified KMI, SAS, and SDS

**Figure 2:**
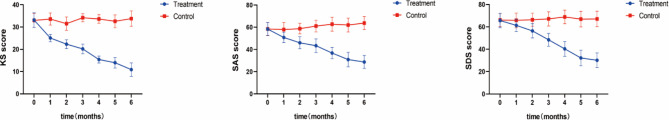
Clinical Symptoms. Measured by the Modified KMI, SAS, and SDS After 5 months of treatment, the KMI score of the treatment group decreased to 13.98, which was close to normal; In the treatment group, the SAS score decreased to 45.98 points after 2 months of treatment, indicating that patients had more or less got rid of anxiety; An SDS score of more than 50 usually indicates a depressive state, while patients in the treatment group fully recovered after 5 months of SHD treatment, with their score dropping to 32.20.

Our primary results showed that the patients in both groups experienced a moderate degree of menopausal symptoms with an average KMI score of approximately 33. After treatment for 5 months, the scores in the treatment group decreased to 13.98, indicating near-normal scores. However, the patients in the control group still presented with moderate stress without any improvements. A score of 50–59 indicated a mild state of anxiety on the SAS. We found that the scores in the treatment group decreased to 45.98 after only 2 months of treatment, which means that patients were more or less free of anxiety. In comparison, the control group demonstrated a continuous state of moderate anxiety until the end of the clinical trial. In China, an SDS score of more than 50 normally indicates a state of depression (**Table 2**). Therefore, our results showed that all patients in the control group experienced a relatively serious depression during the whole trial period; however, the patients in the treatment group recovered completely on the fifth month of SHD treatment with scores decreasing to 32.20. Statistical differences were observed between the two groups after only 1 month of treatment as shown in **Figure 2**.

**Table 2:** Kupperman as well as SAS and SDS scores changes during clinical trial for 6 months (n=45 each group).

Groups	Indices	Start	1 month	2 months	3 months	4 months	5 months	6 months
Treatment	Kupperman Scores	33.14 ± 3.28	25.08 ± 1.65	22.31 ± 1.95	20.18 ± 2.13	15.41 ± 1.57	13.98 ± 2.34	10.87 ± 3.02
Control		32.98 ± 3.16	33.59 ± 2.75	31.52 ± 3.04	34.18 ± 1.78	33.63 ± 1.98	32.57 ± 2.84	33.77 ± 3.45
	P value	0.26	0.04	0.03	0.00	0.00	0.00	0.00
Treatment	SAS	58.32 ± 5.96	50.76 ± 4.65	45.98 ± 5.37	43.37 ± 6.12	36.75 ± 5.18	30.91 ± 6.45	28.72 ± 5.83
Control		58.43 ± 5.78	57.87 ± 6.42	58.72 ± 4.76	61.03 ± 5.18	62.72 ± 6.32	61.97 ± 6.18	63.67 ± 5.89
	P value	0.83	0.04	0.02	0.00	0.00	0.00	0.00
Treatment	SDS	65.73 ± 6.39	61.34 ± 5.48	56.45 ± 6.32	48.42 ± 5.72	40.38 ± 6.59	32.20 ± 7.03	30.18 ± 6.52
Control		66.12 ± 5.87	65.87 ± 6.52	66.37 ± 5.84	67.18 ± 6.34	68.92 ± 6.18	66.98 ± 7.02	67.13 ± 6.98
	P value	0.59	0.04	0.03	0.00	0.00	0.00	0.00

### Laboratory Data Results

**Figure 3:**
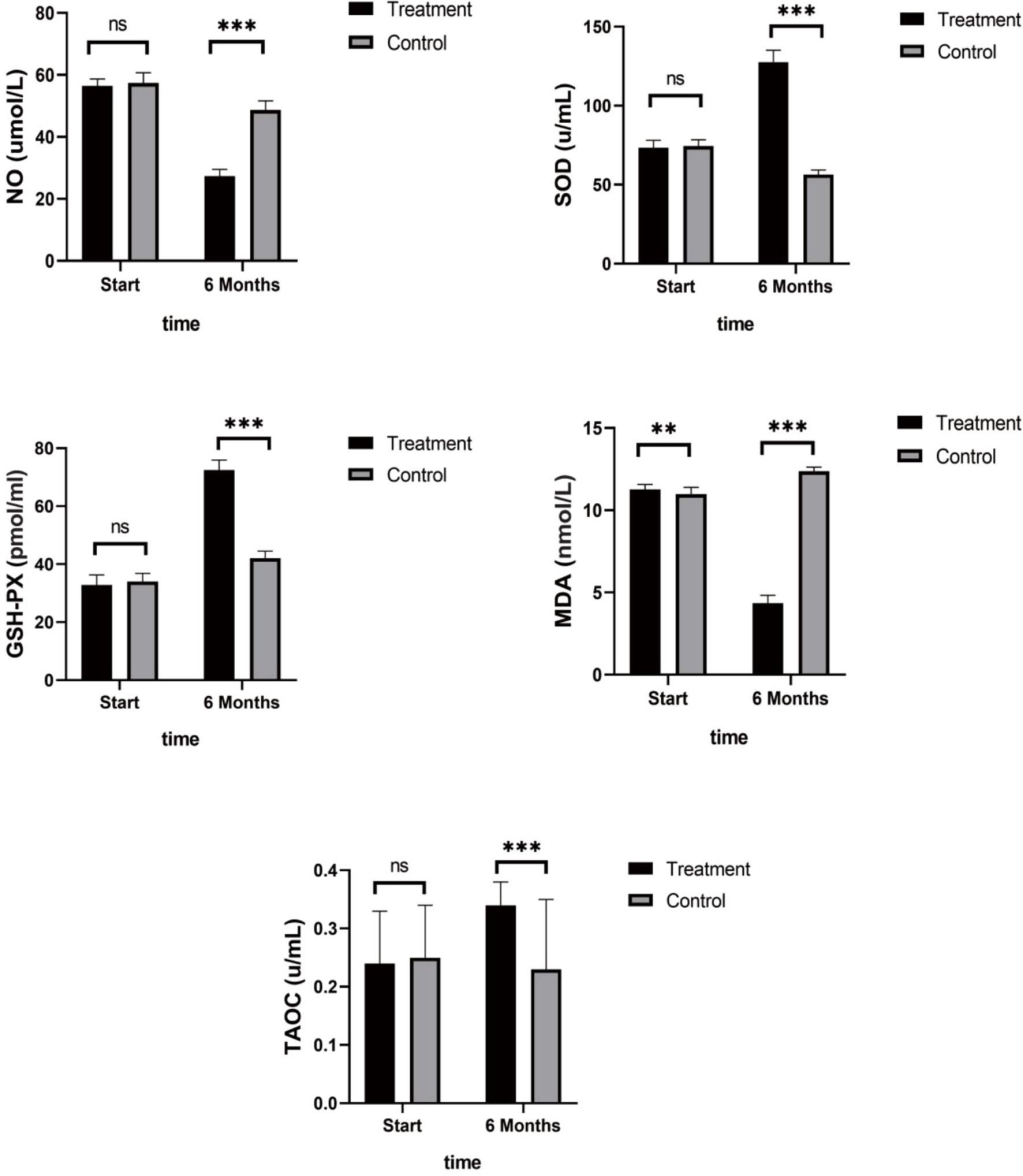
Antioxidant Indices. Patients in the control group showed stable or increased NO and MDA levels, slight decreases in the anti-stress factor, SOD, and stable levels of TAOC and GSH-PX. While the treatment group showed stress-promoting factor levels that had decreased to approximately 30% to 50% and anti-stress factor levels that had increased between 50% and 200%. (ns mean non-statistics significance **P ≤ 0.005; ***P ≤ 0.001).

Patients in the control group who only received endocrine therapy for 6 months showed stable or increased NO and MDA levels, slight decreases in the anti-stress factor, SOD, and stable levels of TAOC and GSH-PX (**Table 3**). In contrast, the patients in the treatment group showed stress-promoting factor levels that had decreased to approximately 30% to 50% and anti-stress factor levels that had increased between 50% and 200%. Significant differences were observed between the two groups. Detailed data are shown in **Figure 3**.

**Table 3:** Changes of stress related serum factors during 6 months of treatment (n=45 each group).

Groups	NO (umol/L)		SOD (u/ml)		GSH-PX (pmol/ml)	
	Start	6 months	Start	6 months	Start	6 months
Treatment	56.42 ± 2.31	27.39 ± 2.14	73.45 ± 4.63	127.59 ± 7.52	32.81 ± 3.51	72.43 ± 3.46
Control	57.42 ± 3.28	48.73 ± 2.89	74.48 ± 3.97	56.42 ± 2.92	34.05 ± 2.8	42.07 ± 2.45
P value	0.15	0.00	0.91	0.00	0.23	0.00
**Groups**	**MDA (nmol/L)**		**TAOC (u/ml)**			
	**Start**	**6 months**	**Start**	**6 months**		
Treatment	11.25 ± 0.32	4.36 ± 0.47	0.24 ± 0.09	0.34 ± 0.04		
Control	10.98 ± 0.41	12.37 ± 0.25	0.25 ± 0.09	0.23 ± 0.12		
P value	0.004	0.00	0.95	0.00		

**Figure 4:**
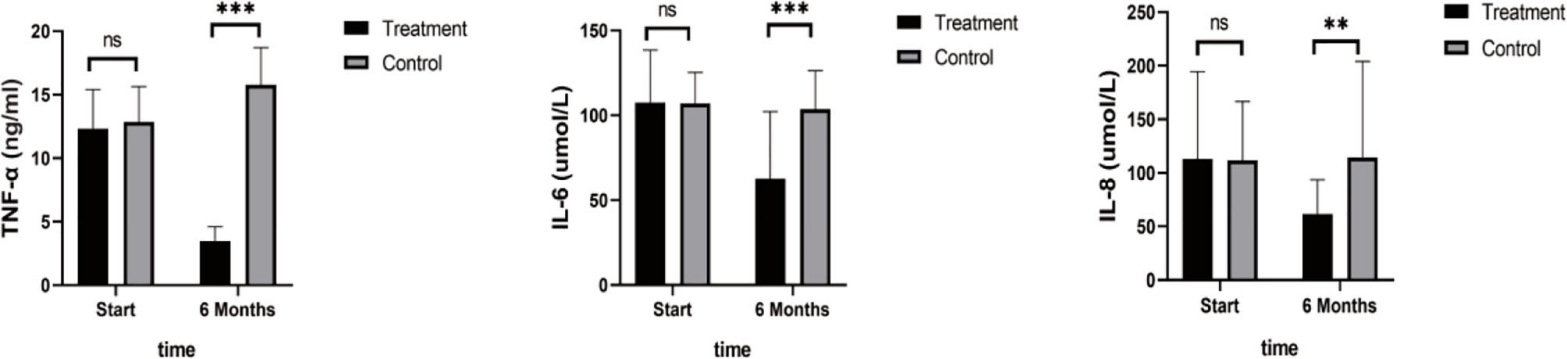
Inflammatory Factors. Compared with the control group, TNF-α, IL-6, and IL-8 were significantly reduced after 6 months in the SHD group. (ns mean non-statistics significance **P ≤ 0.005; ***P ≤ 0.001).

**Figure 5:**
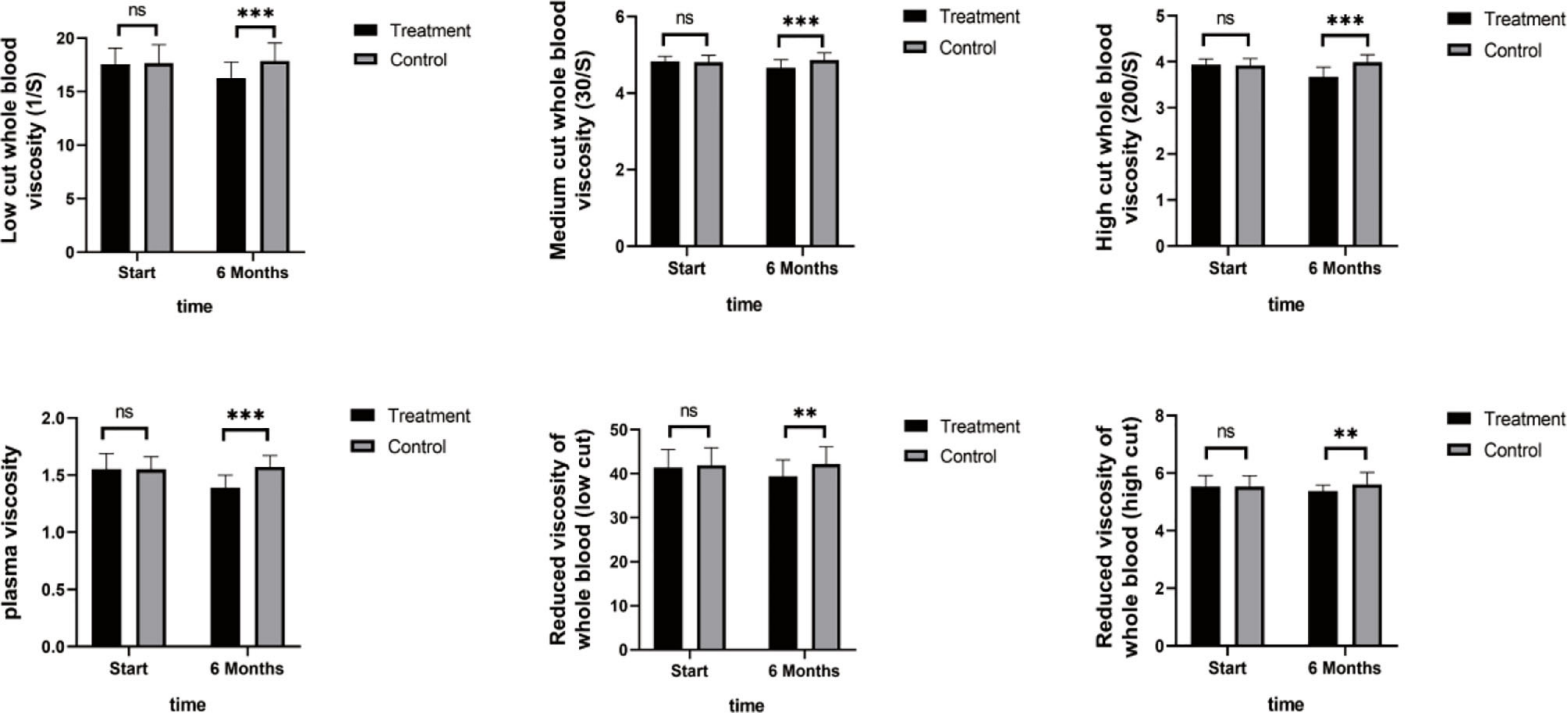
Hemorheology Study. The study of plasma and whole blood viscosity showed that plasma viscosity, whole blood viscosity (high cut), and whole blood viscosity (low cut) were significantly reduced compared with the control group, with plasma viscosity being most significantly reduced. (ns mean non-statistics significance **P ≤ 0.005; ***P ≤ 0.001).

**Figure 6:**
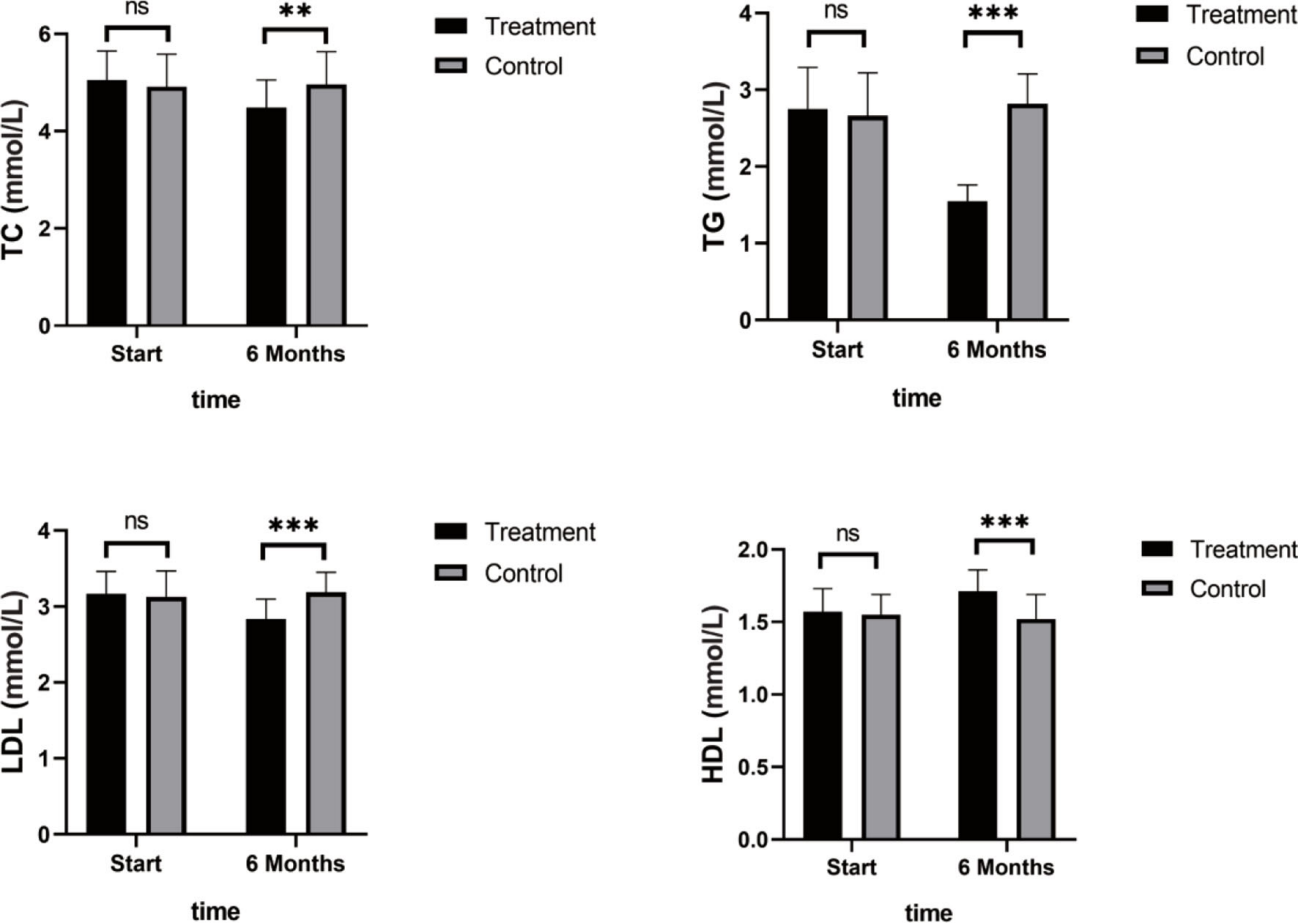
Blood Lipid Study. TG, TC, and LDL levels decreased significantly in the treatment group, but HDL levels increased significantly. (ns mean non-statistics significance **P ≤ 0.005; ***P ≤ 0.001).

**Figure 7:**
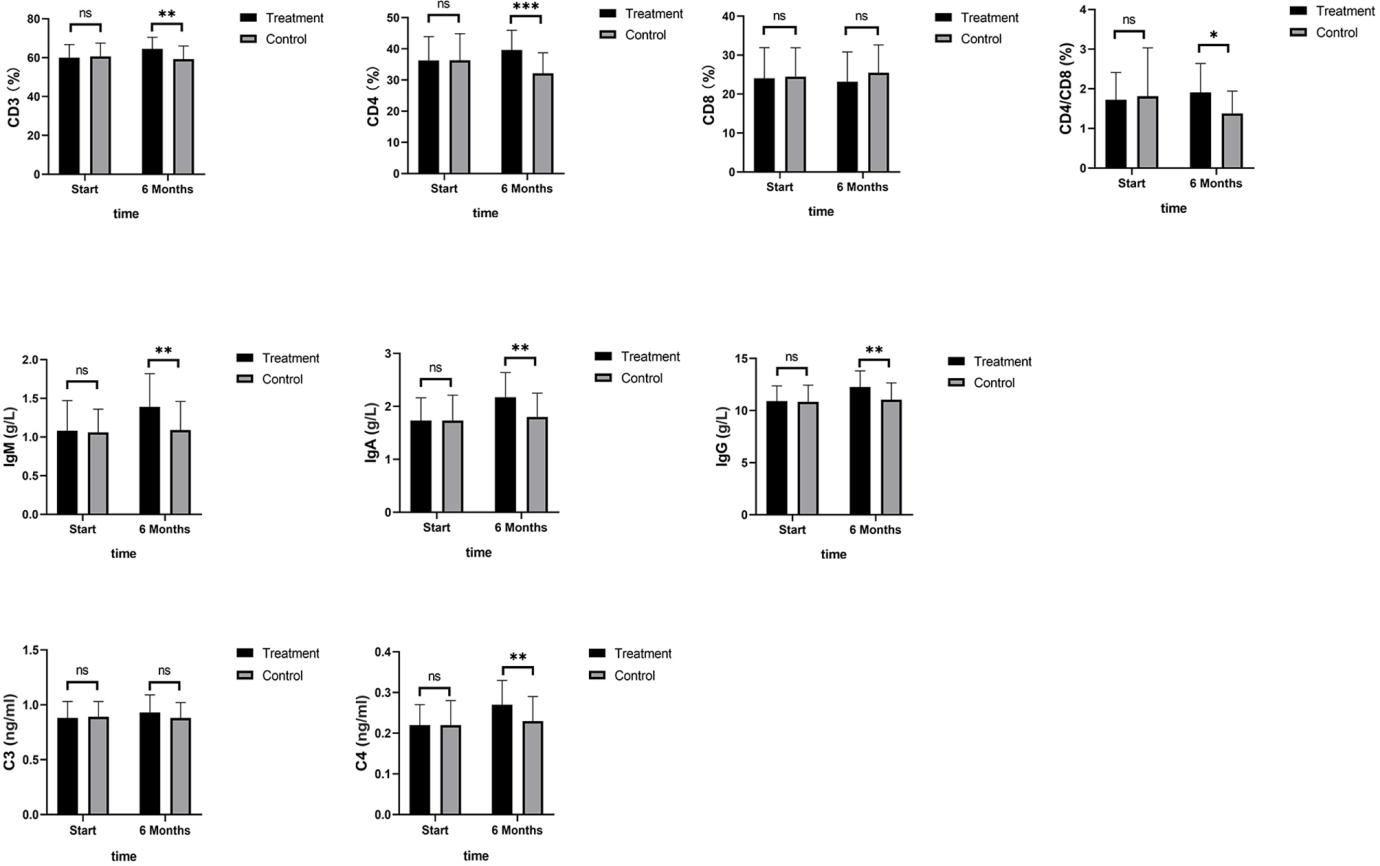
Immune Factors. The immune factor (CD8, and C3) was not statistically significant compared with the control group after 6 months of SHD treatment; The indices of tumor cell proliferation inhibition (CD3, CD4, IgM, IgA, IgG and C4) were significantly improved after 6 months of SHD treatment. CD4/CD8 had improved compared with the control group. (ns mean non-statistics significance *P ≤ 0.05; **P ≤ 0.005; ***P ≤ 0.001).

As shown in **Figure 4**, TNF-α, IL-6, and IL-8 were significantly reduced after 6 months in the SHD group, but not in the control group, and this indicated that SHD can significantly improve the inflammatory response of patients with breast cancer treated with endocrine therapy. The hemorheology study showed that low cut whole blood viscosity (1/S), medium cut whole blood viscosity (30/S), and high cut whole blood viscosity (200/S) were significantly reduced compared with the control group. The largest significant difference was for high cut whole blood viscosity (200/S). Similarly, the study of plasma and whole blood viscosity showed that plasma viscosity, whole blood viscosity (high cut), and whole blood viscosity (low cut) were significantly reduced compared with the control group, with plasma viscosity being most significantly reduced (**Figure 5**). The results of the blood lipid study showed that TG, TC, and LDL levels decreased significantly in the treatment group, but HDL levels increased significantly (**Figure 6**). In the study of immune factors, it was found that the immune factors CD8 and C3 were not statistically significant compared with the control group after 6 months of SHD treatment (**Table 4**). However, the indices of tumor cell proliferation inhibition (CD3, CD4, CD4/CD8, IgM, IgA, IgG, and C4) were significantly improved after 6 months of SHD treatment, and CD4/CD8 had improved compared with the control group (**Figure 7**).

**Table 4:** Change levels of inflammatory factors,hemorheology,lipid and immunologic factors in vivo (n=45 each group).

Groups	Inflammatory factors							
	TNF- (ng/ml)		IL-6 (μmol/L)			IL-8 (μmol/L)		
	Start	6 months	Start	6 months		Start		6 months
Treatment	12.31 ± 3.1	3.48 ± 1.12	107.39 ± 31.02	62.71 ± 39.47		112.92 ± 81.66		61.67 ± 31.94
Control	12.85 ± 2.8	15.78 ± 2.93	106.91 ± 18.40	103.54 ± 22.74		111.74 ± 54.83		114.58 ± 89.43
P value	0.76	0.00	1.00	0.00		1.00		0.00
Groups	Hemorheological indices (mPa.s)							
	Low cut whole blood viscosity (1/S)		Medium cut whole blood viscosity (30/S)			High cut whole blood viscosity (200/S)		
	Start	6 months	Start		6 months	Start	6 months	
Treatment	17.55 ± 1.5	16.28 ± 1.49	4.83 ± 0.13		4.66 ± 0.21	3.94 ± 0.12	3.67 ± 0.21	
Control	17.65 ± 1.74	17.86 ± 1.68	4.81 ± 0.18		4.86 ± 0.19	3.92 ± 0.15	3.99 ± 0.16	
P value	1.00	0.00	0.95		0.00	0.94	0.00	
Groups	Hemorheological indices (mPa.s)							
	plasma viscosity		Reduced viscosity of whole blood (high cut)			Reduced viscosity of whole blood (low cut)		
	Start	6 months	Start		6 months	Start	6 months	
Treatment	1.55 ± 0.14	1.39 ± 0.11	5.53 ± 0.39		5.37 ± 0.21	41.43 ± 4.06	39.39 ± 3.75	
Control	1.55 ± 0.11	1.57 ± 0.1	5.53 ± 0.38		5.61 ± 0.41	41.94 ± 3.95	42.18 ± 3.95	
P value	0.99	0.000	0.99		0.009	0.93	0.005	
Groups	Lipid indices (mmol/L)							
	TC		TG			LDL		
	Start	6 months	Start		6 months	Start	6 months	
Treatment	5.05 ± 0.6	4.48 ± 0.57	2.75 ± 0.54		1.55 ± 0.21	3.17 ± 0.29	2.84 ± 0.26	
Control	4.91 ± 0.67	4.96 ± 0.67	2.66 ± 0.56		2.82 ± 0.39	3.13 ± 0.34	3.19 ± 0.26	
P value	0.72	0.002	0.78		0.000	0.91	0.00	
Groups	Lipid indices (mmol/L)		T cell immunologic indices (%)					
	HDL		CD3			CD4		
	Start	6 months	Start		6 months	Start	6 months	
Treatment	1.57 ± 0.16	1.71 ± 0.15	59.94 ± 6.85		64.47 ± 6.00	36.29 ± 7.59	39.59 ± 6.36	
Control	1.55 ± 0.14	1.52 ± 0.17	60.6 ± 6.91		59.2 ± 6.81	36.33 ± 8.51	32.13 ± 6.61	
P value	0.93	0.00	0.97		0.001	0.99	0.00	
Groups	T cell immunologic indices (%)					B cell immunologic indices		
	CD8		CD4/CD8			IgM (g/L)		
	Start	6 months	Start		6 months	Start	6 months	
Treatment	24.06 ± 7.85	23.18 ± 7.64	1.72 ± 0.69		1.91 ± 0.73	1.08 ± 0.39	1.39 ± 0.43	
Control	24.47 ± 7.42	25.47 ± 7.16	1.81 ± 1.22		1.38 ± 0.56	1.06 ± 0.3	1.09 ± 0.37	
P value	0.99	0.47	0.96		0.016	0.99	0.001	
Groups	B cell immunologic indices (g/L)					Complement indices (ng/ml)		
	IgA		IgG			C3		
	Start	6 months	Start		6 months	Start	6 months	
Treatment	1.73 ± 0.43	2.17 ± 0.47	10.91 ± 1.46		12.25 ± 1.55	0.88 ± 0.15	0.93 ± 0.16	
Control	1.73 ± 0.48	1.8 ± 0.45	10.85 ± 1.6		11.04 ± 1.62	0.89 ± 0.14	0.88 ± 0.14	
P value	0.99	0.001	0.99		0.002	0.99	0.38	
Groups	Complement indices (ng/ml)							
	C4							
	Start	6 months						
Treatment	0.22 ± 0.05	0.27 ± 0.06						
Control	0.22 ± 0.06	0.23 ± 0.06						
P value	0.99	0.007						

### Biomarkers

**Figure 8:**
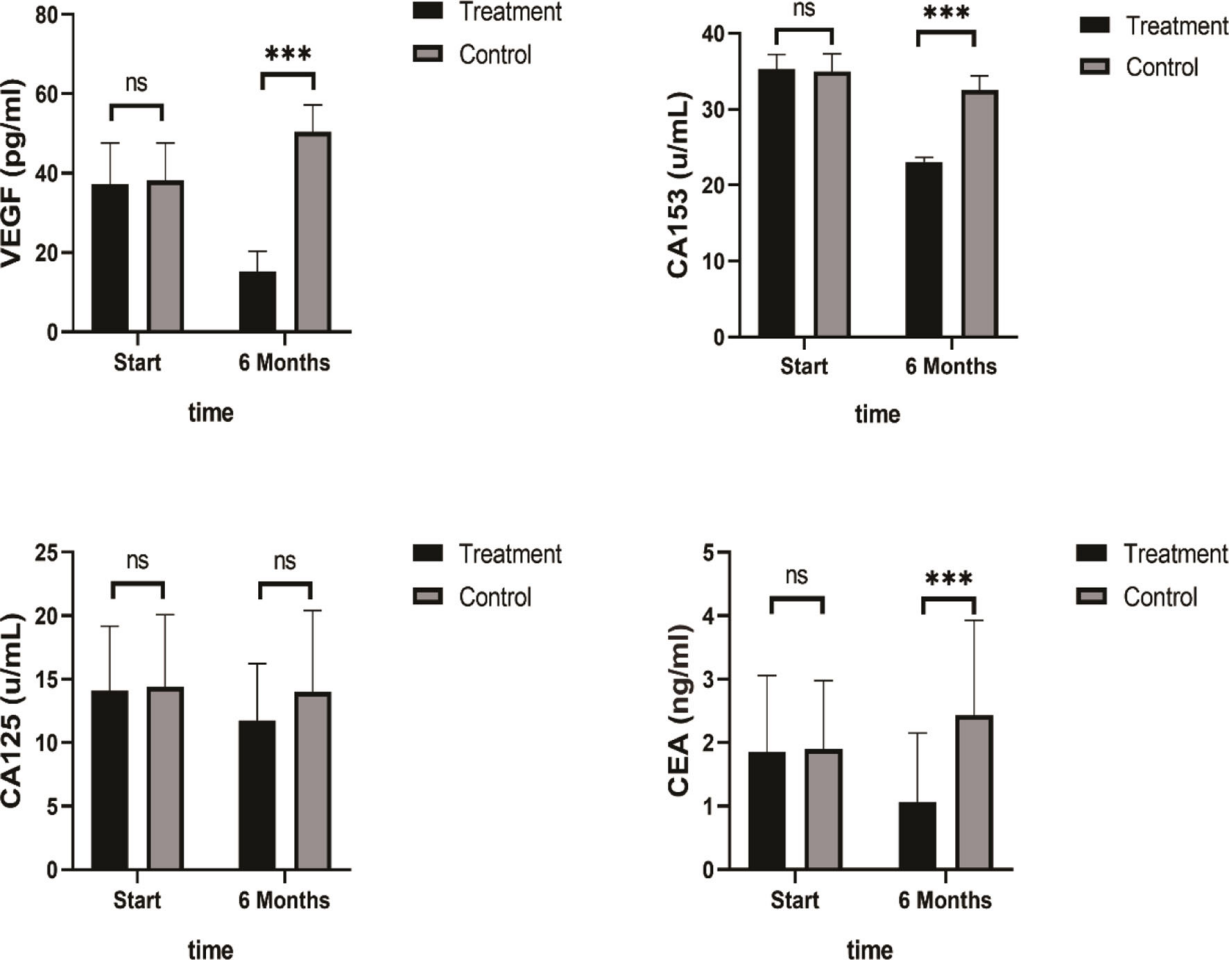
Tumor Biomarkers VEGF, CA153, and CEA levels decreased significantly after 6 months of SHD treatment, but CA125 had non-statistics significance. (ns mean non-statistics significance ***P ≤ 0.001).

A summary of the findings of the tumor growth-related biomarkers at baseline and after 6 months of SHD treatment is presented in **Table 5**. VEGF, CA153, and CEA levels decreased significantly after 6 months of SHD treatment (P < 0.01), illustrating that endocrine treatment combined with SHD can significantly improve the curative effect and may provide a better prognosis for patients with breast cancer. But, CA125 had a non-statistical significance (**Figure 8**).

**Table 5:** Changes of VEGF,CA153,CA125 and CEA.

Groups	VEGF (pg/ml)		CA153 (u/ml)		CA125 (u/ml)	
	Start	6 months	Start	6 months	Start	6 months
Treatment	37.28 ± 10.34	15.14 ± 5.25	35.28 ± 1.92	23.08 ± 0.57	14.12 ± 5.05	11.75 ± 4.49
Control	38.14 ± 9.47	50.43 ± 6.82	34.96 ± 2.34	32.54 ± 1.87	14.40 ± 5.67	14.00 ± 6.41
P value	0.96	0.00	0.83	0.00	0.99	0.21
Groups	CEA (ng/ml)					
	Start	6 months				
Treatment	1.85 ± 1.21	1.06 ± 1.09				
Control	1.90 ± 1.08	2.43 ± 1.50				
P value	1.00	0.00				

## Discussions

Our results showed a statistically significant improvement in all three scores after only 1 month of oral administration of SHD. However, after treatment for 6 months, the KMI score was reduced to one-third of the baseline scores before treatment, while the scores of SDS and SAS reduced to half of the baseline scores. It has been reported that the KMI score could be reduced by 63% with estrogen replacement treatment without increasing the risk of recurrence in the short-term. Our results achieved effects comparable to that of estrogen replacement treatment, with a decrease of 67%. Some clinical trials have reported that lifestyle and emotions, such as worry and fear, may have a positive influence on the prognosis of breast cancer (27, 28). However, here, we report relief from chronic stress caused by long-term endocrine treatment with the use of oral SHD, which facilitates a positive change in the tumor microenvironment.

At baseline, nearly 70% of the patients recruited in the study were in the early stage of breast cancer with no lymph node metastasis. Therefore, it was expected that they would have a long lifespan and a normal lifestyle. Moreover, they may have had a high demand for the quality of life and paid more attention to the feeling of being unwell. More than 90% of patients started endocrine treatment within one and a half years, which meant that the uncomfortable feelings, such as gastrointestinal reaction, irregular menstruation, et al., may be the result of the sudden adaptation to endocrine treatment within a short period of time.

In contrast to most clinical studies, which have applied psychological regulation interventions, such as mindfulness, self-education, or anti-oxidative food intake to relieve chronic stress, we employed a TCM decoction. The hospital pharmacy posted a drug-decocting machine directly to the home address of the patient, along with standard boiling procedure instructions. This made it easy for patients to administer SHD, thereby enhancing compliance to ensure clinical effects.

Due to the health care insurance policies in China, patients only receive 1 month of medication at a time and have to attend the hospital once a month for the next. Therefore, it was convenient for us to evaluate the KMI, SDS, and SAS once a month.

The KMI, SAS, and SDS scores provide information about the multiple dimensions of the status of patients during endocrine treatment. Previous studies have reported that symptoms of depression and anxiety remain unchanged throughout the course of endocrine treatment (29). Our results for the control group showed similar results, indicating that patients may experience continual chronic stress; however, we also showed that this condition could be relieved with TCM treatment in 1 month.

Studies have shown that uncomfortable feelings experienced during endocrine treatment are closely related to chronic stress (30, 31). It was reported that variations in experience were related to emotional distress in women undergoing endocrine treatment, suggesting the importance of including an assessment of chronic stress to fully understand the extent of stress-depression relationships and the underlying mechanisms. Thus, we evaluated the serum changes related to oxidative and anti-oxidative stress during 6 months of endocrine treatment. In accordance with the amelioration of emotional state and quality of life, the serum indices related to stress improved significantly after treatment with SHD.

Long-term chronic stress in breast cancer patients has been reported to be closely related to the progress, metastasis, and prognosis of such patients (32). Mechanical research has shown that stress increases therapy resistance through epithelial-mesenchymal transition markers and promotes lung metastatic colonization of circulating breast cancer cells by creating a pre-metastatic niche by activating β-adrenergic signaling (33). In addition, when patients are treated with long-term endocrine therapy, resistance to endocrine therapy may be mediated in part by ROS-mediated dysregulation of redox-sensitive signaling pathways (34). Our results also showed that the sera of patients who received endocrine treatment for 6 months was not able to inhibit the growth of tamoxifen-resistant cells, while such sera could acquire this inhibitory ability with a decrease in chronic stress through the administration of oral SHD.

Many factors are closely related to chronic stress. Extensive research during the last two decades has revealed the mechanism by which continued oxidative stress can lead to chronic inflammation (35). There are reports that chronic stress could induce a microenvironment with an enhanced expression of inflammatory factors, such as TNF-α, IL-1, IL-6, and IL-8, which are believed to play a role in malignant tumor progression and negative prognosis in cancers, including breast cancer (36, 37). It was previously reported in a clinical trial that breast cancer patients may enjoy a good prognosis by reducing the serum levels of inflammatory factors IL-6, IL-8, and TNF-α, and this might even offer protection from the metastases and recurrence of breast cancer (38). Our results showed that the serum levels of such factors could be reduced to approximately 30% to 50% of baseline levels in breast cancer patients treated with SHD for chronic stress. Our results remind us that a continued interference with the state of stress may provide a preferred inflammatory microenvironment for tumor growth and progression.

It has been reported that long-term continued endocrine treatment, which results in estrogen deprivation and loss of its protective function, results in a high incidence of hypercholesterolemia, which ultimately leads not only to high risk of cardiovascular disease but also an even higher risk of mortality. It was reported that the addition of blood lipid control treatment would effectively improve the disease-free survival in such patients (38). Our research showed that treatment with TCM cannot only improve the quality of life during endocrine treatment but also reduce the serum levels of blood lipids, thus producing a favorable environment for body recovery and relief from the side effects of endocrine treatment simultaneously.

Since research has reported that cellular and humoral immunodeficiency, which may be caused by stress and depression in breast cancer patients, could produce resistance to hormone therapy and often correlates with a poor prognosis, we checked these indices during the 6-month endocrine treatment period and found that CD4 was inhibited and the tumor growth markers, VEGF and CEA, levels improved. The results are partly in accordance with those of other reports that showed significantly increased plasma VEGF levels. Our results revealed that SHD could effectively regulate the cellular and humoral immunity state during endocrine treatment and significantly downregulate the tumor growth markers, VEGF and CEA.

Research suggests that higher levels of bodily stress can predict patient relapse in high-risk ER(+) breast cancer patients receiving endocrine therapy (39). Moreover, mitochondrial markers, which represent the oxidative stress state of the body, are closely related to tumor recurrence, metastasis, and tamoxifen resistance (40). Extensive research over the last two decades has suggested that oxidative stress, chronic inflammation, and cancer are closely linked. Several inflammatory mediators, such as TNF-α, IL-6, TGF-β, and IL-10, have been shown to play a role in cancer progression (41). In this study, we explored the levels of some of these cytokines in the tumor microenvironments in terms of their capacity to generate reactive oxygen and nitrogen species, and their potential involvement in the mechanisms of angiogenesis and drug resistance. As previously reported, SHD downregulates aurora kinase A to inhibit breast cancer cell growth and ameliorate inflammatory status in breast cancer patients during the perioperative period. In addition, emodin, as the main ingredient of SHD, inhibits the bioactivity of tamoxifen-resistant breast cancer cells by inhibiting the PI3K pathway and inhibiting angiogenesis.

However, there had been some limitations in our research. The patients included in our clinical trial had an average age of 51–53 years, and most would have been experiencing perimenopause and possibly discomfort induced by a significant estrogen reduction. In addition, all recruited patients underwent mastectomy with or without axillary lymphadenectomy, which would increase the psychological stress due to the deformed chest shape with one breast missing. Furthermore, 6 months of endocrine treatment would produce additional discomfort, which may exacerbate the stress even further. Such factors experienced together could have a moderate influence on the responses of the patients on the KMI, as well as the SAS and SDS, and these symptoms would not have been relieved if treatment measurements had not been undertaken.

In summary, our clinical research showed that the chronic stress state could be effectively relieved with SHD treatment by controlling the tumor microenvironment with amelioration of chronic oxidative stress in the body. SHD does this *via* the regulation of stress-related factors, as well as inflammatory, hemorheology, lipid, immune, and angiogenic factors; all of which function to promote tumor growth or drug resistance. SHD treatment was also associated with a significantly improved quality of life, with the patients mainly presenting with optimistic attitudes toward the disease and daily life and a relatively cheerful mood. After 6 months of observation, some tumor biomarkers decreased and this inhibited the growth of tamoxifen-resistant cells. In the future, we would like to expand the clinical research to include a large number of patients and a long observation time to further confirm the advantages of SHD in creating a favorable microenvironment for adjuvant control of breast cancer recurrence and metastasis.

## Data Availability Statement

The raw data supporting the conclusions of this article will be made available by the authors, without undue reservation.

## Ethics Statement

The studies involving human participants were reviewed and approved by The Ethics Committee of Nanjing University of Chinese Medicine. The patients/participants provided their written informed consent to participate in this study.

## Author Contributions

MF and CY designed the experiments. MF, HW, ZZ, SC, and YX performed the experiments. MF analyzed the experimental results. HW and YX analyzed the experimental data. MF and HW wrote the manuscript. MF, CY, BY, and YL reviewed and edited the manuscript. All authors contributed to the article and approved the submitted version.

## Funding

This study was supported by a grant from the Ministry of Health & Welfare, Republic of Korea in 2015 (Grant Number 090-091-3000-3038-301-320-01) and a project funded by the Priority Academic Program Development of Jiangsu Higher Education Institutions, as well as the peak of talent project of Jiangsu Provincial Hospital (TCM-012062003010).

## Conflict of Interest

The authors declare that the research was conducted in the absence of any commercial or financial relationships that could be construed as a potential conflict of interest.

## Publisher’s Note

All claims expressed in this article are solely those of the authors and do not necessarily represent those of their affiliated organizations, or those of the publisher, the editors and the reviewers. Any product that may be evaluated in this article, or claim that may be made by its manufacturer, is not guaranteed or endorsed by the publisher.
